# Genome-wide analysis of the pentatricopeptide repeat gene family in different maize genomes and its important role in kernel development

**DOI:** 10.1186/s12870-018-1572-2

**Published:** 2018-12-19

**Authors:** Lin Chen, Yong-xiang Li, Chunhui Li, Yunsu Shi, Yanchun Song, Dengfeng Zhang, Yu Li, Tianyu Wang

**Affiliations:** Institute of Crop Sciences, Chinese Academy of Agricultural Sciences, Beijing, 100081 China

**Keywords:** Pentatricopeptide repeat (PPR) proteins, Maize, Gene structure, Expression variation, Kernel development

## Abstract

**Background:**

The pentatricopeptide repeat (PPR) gene family is one of the largest gene families in land plants (450 PPR genes in *Arabidopsis*, 477 PPR genes in rice and 486 PPR genes in foxtail millet) and is important for plant development and growth. Most PPR genes are encoded by plastid and mitochondrial genomes, and the gene products regulate the expression of the related genes in higher plants. However, the functions remain largely unknown, and systematic analysis and comparison of the PPR gene family in different maize genomes have not been performed.

**Results:**

In this study, systematic identification and comparison of PPR genes from two elite maize inbred lines, B73 and PH207, were performed. A total of 491 and 456 PPR genes were identified in the B73 and PH207 genomes, respectively. Basic bioinformatics analyses, including of the classification, gene structure, chromosomal location and conserved motifs, were conducted. Examination of PPR gene duplication showed that 12 and 15 segmental duplication gene pairs exist in the B73 and PH207 genomes, respectively, with eight duplication events being shared between the two genomes. Expression analysis suggested that 53 PPR genes exhibit qualitative variations in the different genetic backgrounds. Based on analysis of the correlation between PPR gene expression in kernels and kernel-related traits, four PPR genes are significantly negatively correlated with hundred kernel weight, 12 are significantly negatively correlated with kernel width, and eight are significantly correlated with kernel number. Eight of the 24 PPR genes are also located in metaQTL regions associated with yield and kernel-related traits in maize. Two important PPR genes (GRMZM2G353195 and GRMZM2G141202) might be regarded as important candidate genes associated with maize kernel-related traits.

**Conclusions:**

Our results provide a more comprehensive understanding of PPR genes in different maize inbred lines and identify important candidate genes related to kernel development for subsequent functional validation in maize.

**Electronic supplementary material:**

The online version of this article (10.1186/s12870-018-1572-2) contains supplementary material, which is available to authorized users.

## Background

Since pentatricopeptide repeat (PPR) proteins were discovered and reported in *Saccharomyces cerevisiae* L. [1], PPR genes have been identified and analysed in multiple organisms. The PPR gene family in land plants is very large; for example, 441 members are present in *Arabidopsis*, 477 in rice and 486 in foxtail millet [2–4]. A majority of PPR genes have been confirmed to have functions in plant growth and development, and these genes can affect cytoplasmic male sterility [5–7], embryogenesis [8, 9], and seed development [10–13].

The typical protein sequences found in PPR family members contain multiple tandem arrays of a 35-amino acid PPR domain [2]. The PPR family can be divided into two subfamilies according to the structure of the repeated PPR domain: P and PLS [2, 14]. In addition, PLS subfamilies can be further divided into four subgroups (PLS, E, E+ and DYW) based on different C-terminal motifs [2, 3, 14].

In maize, CRP1, which was the first PPR protein identified, is involved in the translation of the chloroplast *petA* and *petD* mRNAs [15]. The *crp1* mutant does not produce petA and petD proteins, which are important components of cytochrome complex B6F in chloroplasts, because the corresponding polycistronic precursor mRNAs cannot be edited [16]. Many PPR genes in maize exhibit RNA-binding activity and are implicated in mRNA editing in chloroplasts and mitochondria. Recent research has found that the PPR genes not only play a key role maintaining organelle stability but also participate in maize kernel development [11–13, 17, 18]. The mutation *emp 5* (*empty pericarp5*), which encodes a PPR-DYW subgroup protein, results in abortion of the embryo and endosperm in maize [19]. SK1 (*Small kernel 1*) encodes a PPR-E subgroup protein involved in complex I assembly in the mitochondria and, therefore, in kernel development in maize [20]. These studies have helped in identifying the molecular mechanisms underlying PPR gene regulation in the growth and development of maize.

Although the PPR gene family has been identified in the maize inbred line B73 [21], systematic analysis and comparison of this family in different maize genomes have not been performed. Many studies of the diversity of maize have revealed numerous copy number variations (CNVs) and presence/absence variations (PAVs) in the genomes of different inbred lines, especially those from different heterotic groups [22–24]. Fortunately, completion of the genome sequencing of B73 and PH207 provides an excellent opportunity to systematically analyse the PPR gene family in two lines, i.e., B73 and PH207, which represent the stiff stalk heterotic and Iodent heterotic groups in maize, respectively [25, 26].

Here, we present and compare detailed information on the genomic locations and structures, chromosomal distribution, and phylogenetic relationships of the PPR gene family in the B73 and PH207 genomes. In addition, we examine the expression levels of the PPR gene family in these two inbred lines and conduct correlation analysis between the expression of PPR genes and kernel-related traits. Our findings will provide useful information for future research on the molecular mechanisms and biological functions of maize PPR genes.

## Results

### Identification of PPR-encoding genes in the B73 and PH207 genomes

A total of 491 and 456 PPR genes were identified in the B73 and PH207 genomes, respectively, in this study (Table 1). The physical locations, reading frame lengths and protein lengths of these genes are listed in Additional file 1: Table S1 and Additional file 2: Table S2.

**Table 1:** Number of genes in different subgroups of the PPR family in the B73 and PH207 genomes

Genome	P subfamily	PLS subfamily				Total
		E	E+	DYW	PLS	
B73	256	85	48	74	28	491
PH207	251	85	41	67	12	456

**Fig. 1:**
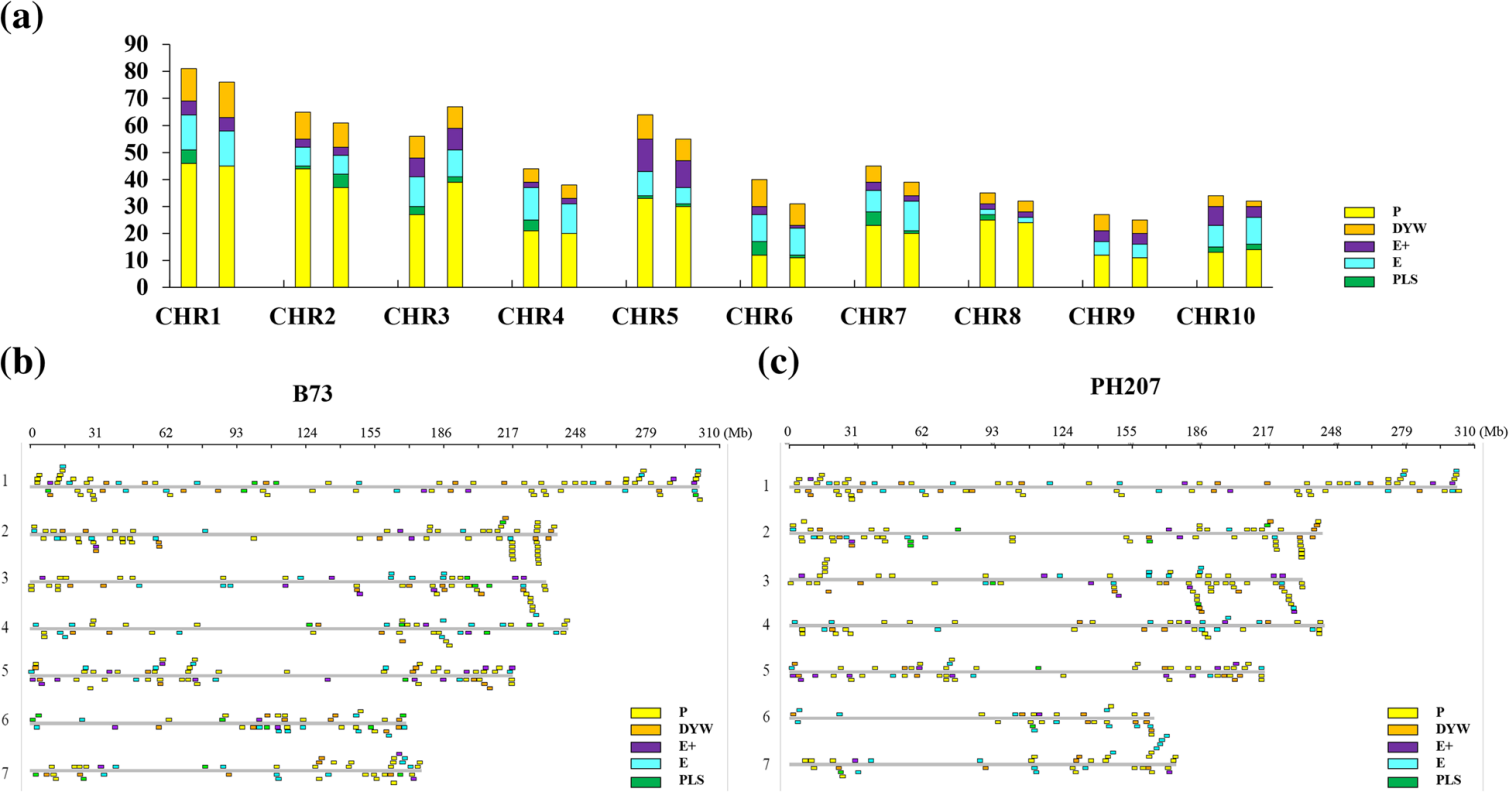
Numbers of genes in different subclasses and distribution of the PPR genes. **a** Numbers of genes in the five subclasses (P, E+, E, DYW, PLS) on each chromosome in B73 and PH207. The left column represents the B73 genome, and the right column represents the PH207 genome. **b** Genomic distribution of PPP genes in the B73 genome. The X axis represents the physical location. **c** Genomic distribution of the PPR genes in the PH207 genome. The X axis represents the physical location

The PPR gene family could be divided into the P (PPR), PLS (P-L-S, PPR-like S (for short) and PPR-like L (for long)), E, E+ and DYW subgroups according to the repeated domain structure. Table 1 provides details of the numbers of PPR genes in each subgroup and in the two maize genomes. The largest difference is the number of PPR genes in the PLS subgroup, with 28 PPR genes in B73 but only 12 in PH207 (Table 1, Fig. 1a). In the B73 genome, the shortest PPR protein family is 114 amino acids in length and the longest 1925 amino acids. In the PH207 genome, the shortest PPR protein is only 79 amino acids and the longest 1946 amino acids (Additional file 1: Table S1 and Additional file 2: Table S2). Subcellular localization prediction using the Target P program showed that 144 PPR proteins (95 PPR proteins in B73 and 49 PPR proteins in PH207) are targeted to chloroplasts and 141 PPR proteins (76 PPR proteins in B73 and 65 PPR proteins in PH207) to mitochondria (Additional file 1: Table S1 and Additional file 2: Table S2).

A number of differences in the PPR gene family were found in the B73 (491 PPR genes) and PH207 genomes (456 PPR genes). Previous studies have suggested the presence of many CNV/PAV differences among maize inbred lines [22–24]. Between the B73 and PH207 genomes, numerous structural variants were also observed [26]. Therefore, we inferred that the number differences in the PPR gene family between these two genomes may be caused by these PAVs. There are 1169 genes that are B73 genotype specific; 1545 genes are PH207 genotype specific [26]. Among these genotype-specific genes, we found 10 genes (5 PPR genes in the B73 genome and 5 PPR genes in PH207 genome) that belong to the PPR gene family (Additional file 1: Table S1 and Additional file 2: Table S2). Compared to the B73 genome, a ~ 55-kb absence on chromosome 2 in the PH207 genome caused the loss of two genes, one of which is a PPR gene (AC195825.3_FG001), and a ~ 48-kb presence on chromosome 3 in the PH207 genome produces an extra PPR gene (Zm00008a013482) (Additional file 3: Figure S1). To better understand the differences in the number of the PPR genes between the B73 genome and PH207 genome, a gene-for-gene comparison was conducted based on criteria that included an E-value less than e^− 10^, identity greater than 40%, and coverage more than 60%. Overall, we found 275 PPR genes with only one copy in the corresponding genome, which can be found in B73 and PH207 (Additional file 4: Table S3). Among the 275 PPR genes, 19 from the B73 genome do not belong to the family in the PH207 genome, and 25 from the PH207 genome do not belong to the family in the B73 genome. A total of 172 PPR genes from the B73 genome have more than two copies in the PH207 genome (Additional file 5: Table S4). Among the 172 PPR genes, 11 from the B73 genome do not belong to the family in the PH207 genome. The remaining 69 PPR genes (including 5 PAV genes identified in a previous study) in the B73 genome and 33 (including 5 PAV genes identified in a previous study) in the PH207 genome have no homologous genes in the corresponding genome.

### Gene structure and chromosomal distribution of PPR genes in B73 and PH207

Table 2 shows the intron numbers of the PPR genes in both of the genomes. A total of 283 and 156 PPR genes were predicted to contain no introns, and 98 and 131 PPR genes were predicted to contain only one intron in B73 and PH207, respectively (Table 2). To better understand the PPR gene structure, we conducted exon/intron analysis and found that the number of introns in PPR genes from the P subclass ranged from 0 to 24 in B73 and from 0 to 16 in PH207 (Table 2 and Additional file 6: Figure S2). Compared with the P subclass, the number of introns in the PLS subclass is relatively low (Additional file 6: Figure S2, Additional file 7: Figure S3). However, GRMZM2G327263, which belongs to the P subclass in the B73 genome, contains 24 introns (Table 2 and Additional file 1: Table S1), and Zm00008a017909, which belongs to the E subgroup in the PH207 genome, contains 17 introns (Table 2 and Additional file 2: Table S2).

**Table 2:** Number of PPR genes with different numbers of introns in the B73 and PH207 genomes

No. of introns	P subclass		PLS subclass								Percentage (Total)
			DYW		E		E+		PLS		
	B73	PH207	B73	PH207	B73	PH207	B73	PH207	B73	PH207	
0	134	70	44	23	58	41	32	21	19	1	46.78%
1	56	73	18	19	12	22	9	11	3	6	24.18%
2	21	41	3	8	6	7	3	2	1	1	9.82%
3	7	21	3	8	5	4	1	4	1	1	5.81%
4	7	12	2	7	3	4	2	2	0	1	4.22%
5	11	9	0	1	0	3	0	1	1	2	2.96%
6	6	6	0	1	0	2	1	0	2	0	1.90%
7	2	4	1	0	0	1	0	0	1	0	0.95%
8	3	5	0	0	1	0	0	0	0	0	0.95%
9	0	2	0	0	1	0	0	0	0	0	0.32%
10	2	3	0	0	0	0	0	0	0	0	0.53%
11	3	3	0	0	0	0	0	0	0	0	0.63%
12	1	0	0	0	0	0	0	0	0	0	0.11%
13	0	1	0	0	0	0	0	0	0	0	0.11%
14	0	0	0	0	0	0	0	0	0	0	0.00%
15	1	0	0	0	0	0	0	0	0	0	0.11%
16	2	1	0	0	0	0	0	0	0	0	0.32%
17	0	0	0	0	0	1	0	0	0	0	0.11%
22	1	0	0	0	0	0	0	0	0	0	0.11%
24	1	0	0	0	0	0	0	0	0	0	0.11%

The PPR genes are unevenly distributed on all 10 maize chromosomes in both B73 and PH207 (Fig. 1a). Chromosome 1 exhibits the most PPR genes in both B73 and PH207 (81 and 76, respectively), whereas chromosome 9 presents the fewest genes in both B73 and PH207 (27 and 25, respectively) (Fig. 1a). Every subgroup except the PLS subgroup occurs on all chromosomes of both genomes. Five, four and two PPR genes of the PLS subgroup are located on chromosomes 1, 4, and 8 in the B73 genome, respectively, but the corresponding chromosomes in the PH207 genome contain no PLS subgroup PPR genes (Fig. 1a). Additionally, we did not observe any PLS subgroup PPR genes on chromosome 9 in either genome (Fig. 1a).

All of the PPR genes were physically mapped onto the whole genomes of B73 and PH207 along with every chromosome with PPR genes (Fig. 1b, c). Multiple PPR gene clusters occur in the B73 and PH207 genomes (Fig. 1b, c). There are many differences in the distributions of the PPR genes between the two genomes. Some PPR gene clusters occur in the B73 genome but not in the PH207 genome; for example, one cluster appears on the end of chromosome 1 (~ 300 Mb) in the B73 genome but is not present in the PH207 genome, while one cluster only occurs on chromosome 2 (~ 227.5 Mb) in the B73 genome, along with one cluster on chromosome 4 (~ 183 Mb) and one cluster on chromosome 10 (~ 118 Mb) in the B73 genome. We also found some PPR gene clusters occurring in the PH207 genome but not in the corresponding regions in the B73 genome: for example, one cluster present on chromosome 3 (~ 1.5 Mb) only occurs in the PH207 genome, along with one cluster on the end of chromosome 6 (~ 163 Mb), one cluster on chromosome 7 (~ 167 Mb), and one cluster on chromosome 10 (~ 142 Mb). In addition to these differences, the PPR gene numbers are also different in one cluster that appears in the same regions in the two genomes (Fig. 1b, c). For example, one cluster on chromosome 1 (~ 1.5 Mb) in the B73 genome contains five PPR genes, but the corresponding cluster in the PH207 genome contains only three PPR genes, another cluster on chromosome 2 (~ 216 Mb) in the B73 genome has six PPR genes, while only four PPR genes are present in the PH207 genome; furthermore, the clusters on chromosome 7 (~ 11.5 Mb) and chromosome 8 (~ 167 Mb) in the B73 genome also have different PPR gene numbers compared with the corresponding clusters in the PH207 genome.

### Gene ontology (GO) annotation

**Fig. 2:**
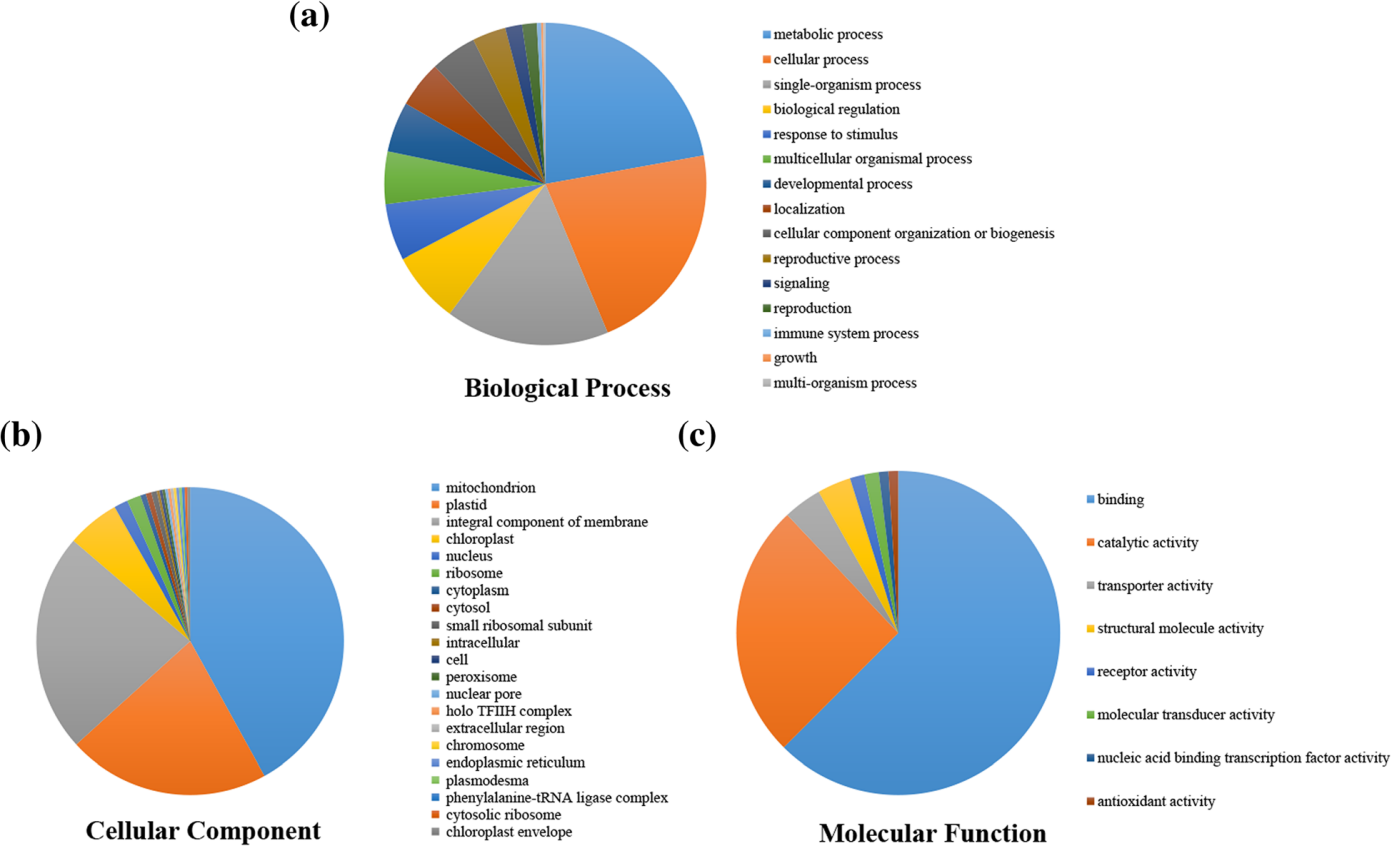
Detailed GO analysis results for maize PPR proteins in B73. Three categories, (**a**) biological process, (**b**) cellular component, and (**c**) molecular function, were identified according to the Blast2GO program

GO analysis suggested the putative participation of PPR genes in multiple biological processes, molecular functions, and cellular components (Fig. 2, Additional file 8: Table S5). Among the 491 PPR genes in B73, GO annotations of 149 genes could not be found, and the other 342 PPR genes were divided among 15 different biological processes (Fig. 2a, Additional file 8: Table S5). GO results indicated that a majority of the PPR genes are likely related to metabolic processes, followed by cellular processes (102, 21.66%), single-organism processes (78, 16.46%), and biological regulation (34, 7.17%). A total of 27 PPR genes are predicted to be involved in responses to stimuli (including responses to stress, abiotic stimuli, and biotic stimuli). Notably, 7 PPR genes are predicted to participate in the reproduction process, which includes seed development; these results agree with previous studies in maize [6–12]. We also found two, one, and one PPR genes are predicted to be involved in immune system processes, growth, and multi-organism processes, respectively. In total, 144 PPR genes are predicted to target mitochondria, 73 to target plastids, 79 to target integral components, and 19 to target chloroplasts (Fig. 2b, Additional file 8: Table S5). Analysis of the molecular functions predicted that 130 PPR genes participate in binding functions, including RNA/DNA binding, RNA editing, and RNA splicing. In addition, the products of 53 PPR genes are predicted to exhibit catalytic activity (Fig. 2c, Additional file 8: Table S5). The results of the biological process and molecular function analyses of the PPR genes in the PH207 genome were consistent with the results from the B73 genome (Additional file 9: Figure S4, Additional file 8: Table S5). Cellular localization analysis results suggested that 144 PPR genes in the B73 genome (134 PPR genes in the PH207 genome) are localized to mitochondria; 19 PPR genes in the B73 genome (14 PPR genes in the PH207 genome) are localized to chloroplasts. These results provide useful information for future gene function studies in maize.

### Motif analysis of PPR proteins in the two genomes

**Fig. 3:**
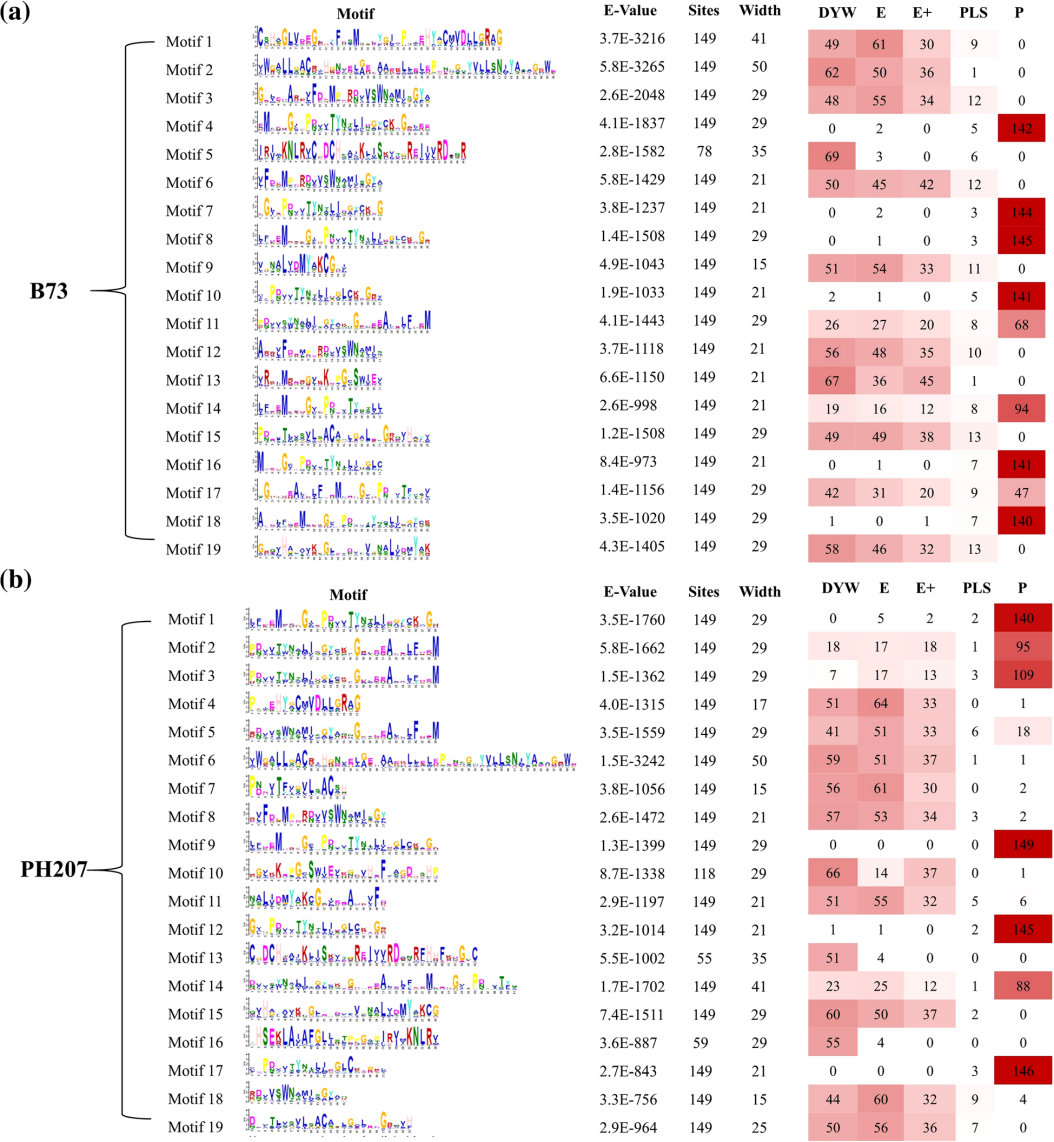
Conserved motifs of PPR proteins in maize. The left panel shows the conserved motif sequence logos, and the right panel shows the number of each motif in the different subgroups of the PPR family in (**a**) the B73 genome and (**b**) the PH207 genome

The motifs of the protein sequences of the PPR gene family in B73 and PH207 were obtained by using MEME Suite (Additional file 6: Figure S2, Additional file 7: Figure S3). A total of 19 motifs were identified in the two genomes (Fig. 3). In the B73 genome, all of the PPR proteins contain Motif 11, Motif 14 and Motif 17, which suggests that all PPR proteins have a highly conserved domain (Fig. 3a). We also found that the different subgroups exhibit several special motifs (Fig. 3a). Six motifs (Motif 4, Motif 7, Motif 8, Motif 10, Motif 16 and Motif 18) were mainly found in the P subgroup, whereas Motif 5 was mainly in the DYW subgroup. The E subgroup did not have Motif 18, and the PLS subgroup contain all the identified motifs.

For PPR proteins in the PH207 genome, 19 motifs were also found, among which eight motifs (Motif 2, Motif 3, Motif 5, Motif 6, Motif 8, Motif 11, Motif 14 and Motif 18) were found in all PPR proteins (Fig. 3b). Motif 9 exists in only the P subgroup, suggesting that this motif is a conserved domain in this subgroup. In addition to Motif 9, we found that Motif 1 and Motif 17 were not present in the DYW subgroup, and Motif 17 was also found in the E subgroup. Comparative analysis with the motif analysis results for PPR proteins in the B73 genome revealed that some motifs are conserved in the two genomes, such as Motif 2 in the B73 genome (Motif 6 in the PH207 genome), Motif 8 in the B73 genome (Motif 9 in the PH207 genome) and Motif 11 in the B73 genome (Motif 2 in the PH207 genome).

### Duplications of PPR genes in the two genomes

**Fig. 4:**
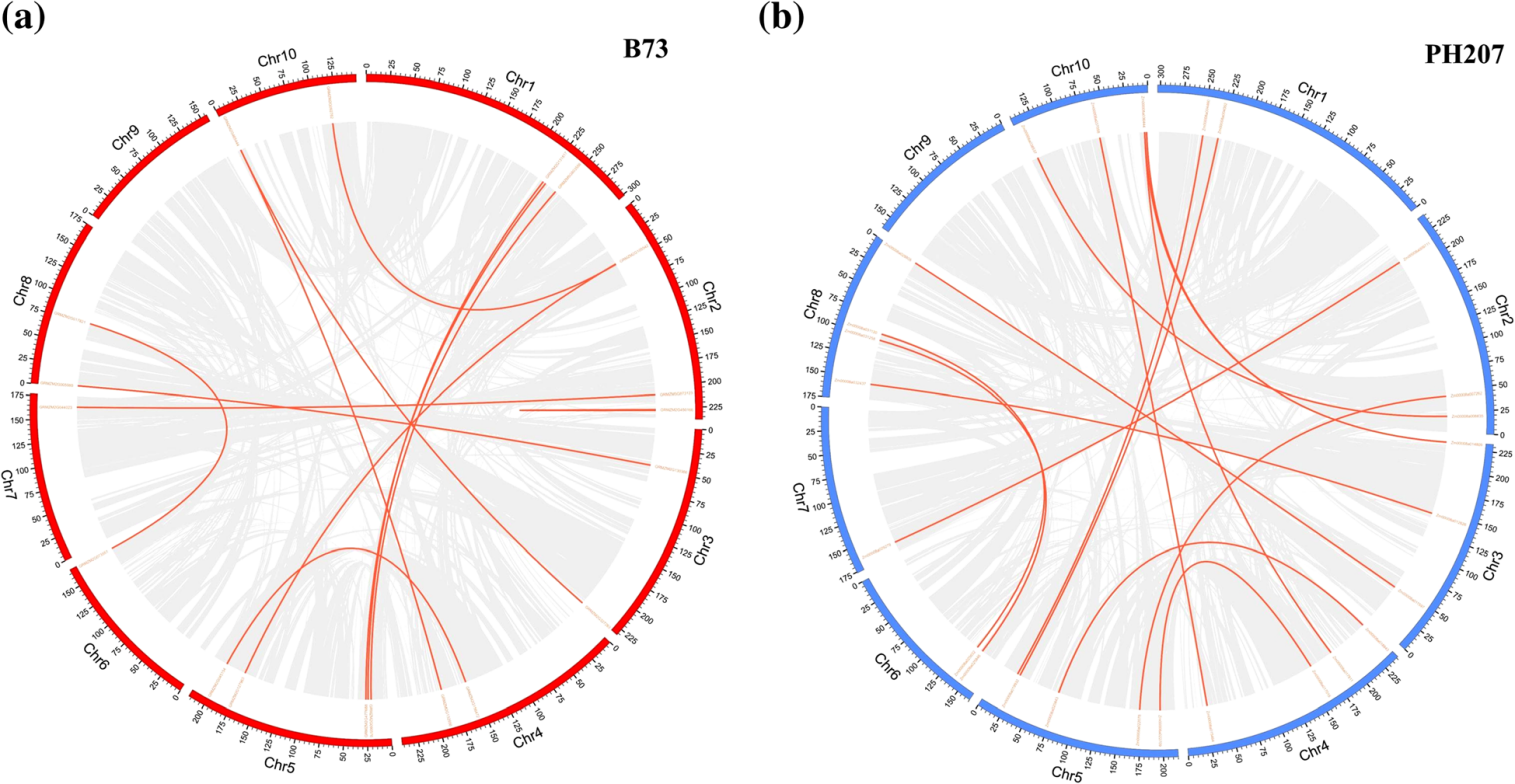
Distribution of segmentally duplicated PPR genes in the maize genome. The grey lines indicate the segmentally duplicated gene pairs in the maize genome, and the red lines indicated the segmentally duplicated PPR gene pairs in (**a**) the B73 genome and (**b**) the PH207 genome

In the B73 genome, we identified 12 segmental duplication gene pairs across the entire genome (Fig. 4a). Only one segmental duplication gene pair, located on chromosome 2, is intra-chromosomal, and the other segmental duplications involve two different chromosomes. Moreover, the duplicated PPR genes belong to the same subgroups (Fig. 4a, Additional file 10: Table S6). The analysis further revealed one special gene duplication involving two genes: GRMZM2G465444, which is located on chromosome 10 and classified in the P subgroup with GRMZM2G327263 on chromosome 3, and GRMZM2G132956 on chromosome 4 (Fig. 4a).

In the PH207 genome, 15 segmental duplication gene pairs were discovered in the PPR gene family (Fig. 4b, Additional file 10: Table S6). In contrast to the duplication events in the B73 genome, all of these duplication events occurred on two different chromosomes. The analysis also revealed one special gene duplication involving different subgroups: Zm00008a018843, which belongs to the P subgroup, and Zm00008a020843, which belongs to the E+ subgroup (Fig. 4b, Additional file 10: Table S6).

Through comparison of the duplication events of PPR genes in the two genomes, we found four and seven special duplication events in B73 and PH207, respectively (Additional file 10: Table S6). Furthermore, we calculated the Ka (the ratio of the number of nonsynonymous substitutions per non-synonymous site) and Ks (the ratio of the number of synonymous substitutions per synonymous site) values of segmental duplication gene pairs in the two genomes and found the Ka/Ks ratios to be lower than 1, indicating that these gene pairs had experienced purifying selection. Additionally, positive selection of the segmentally duplicated gene pairs on chromosome 2 (GRMZM2G450166 vs. GRMZM2G124602) was identified in the B73 genome (Additional file 10: Table S6).

**Fig. 5:**
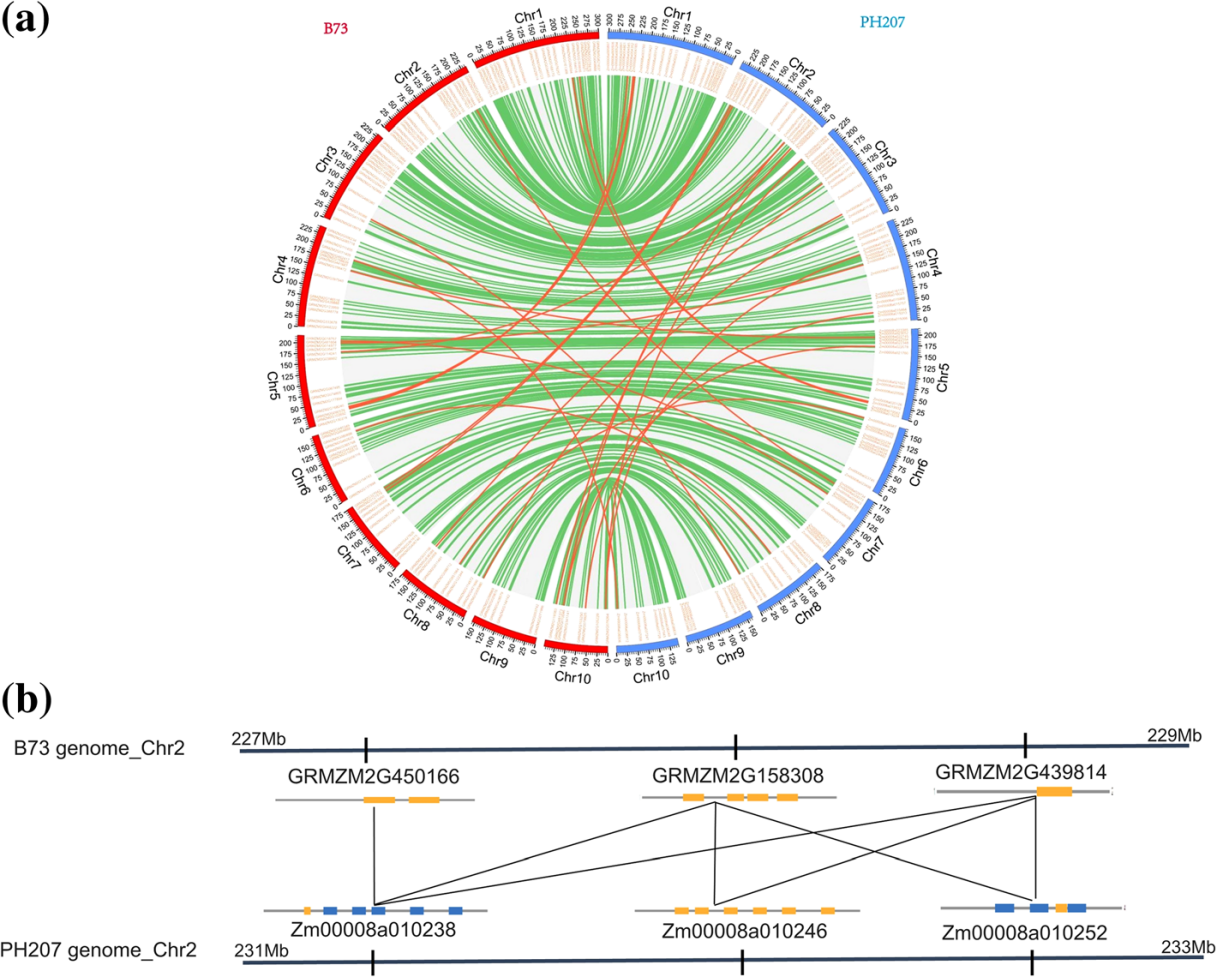
Comparative analysis of PPR genes between the B73 genome and the PH207 genome. **a** Synteny analysis of PPR genes in B73 and PH207. The green lines indicate that these PPR genes are located on the same chromosomes in the two genomes, and the red lines indicate that they are located on different chromosomes. **b** Common paralogs of three PPR genes on chromosome 2 in the B73 genome. GRMZM2G158308 and GRMZM2G439814 on chromosome 2 in the B73 genome have three identical paralogs on the same chromosome of the PH207 genome, and GRMZM2G158308, GRMZM2G439814 and GRMZM2G450166 have a common paralogous gene (Zm00008a010238) in the PH207 genome

Moreover, we constructed a comparative syntenic map of B73 associated with PH207 (Fig. 5a), which showed that 332 paralogs of PPR genes are located at the same chromosomal position in the two genomes (Additional file 11: Table S7). Additionally, 24 paralogs are located on different chromosomes (Additional file 12: Table S8). We also found that GRMZM2G158308 and GRMZM2G439814 on chromosome 2 in the B73 genome present three common paralogs (Zm00008a010238, Zm00008a010246, and Zm00008a010252) in the corresponding region in the PH207 genome. Additionally, Zm00008a010238 in the PH207 genome is the common paralog of three genes (GRMZM2G450166, GRMZM2G158308, and GRMZM2G439814) from the B73 genome (Fig. 5b).

### Expression variation in the PPR gene family in the two genomes

**Fig. 6:**
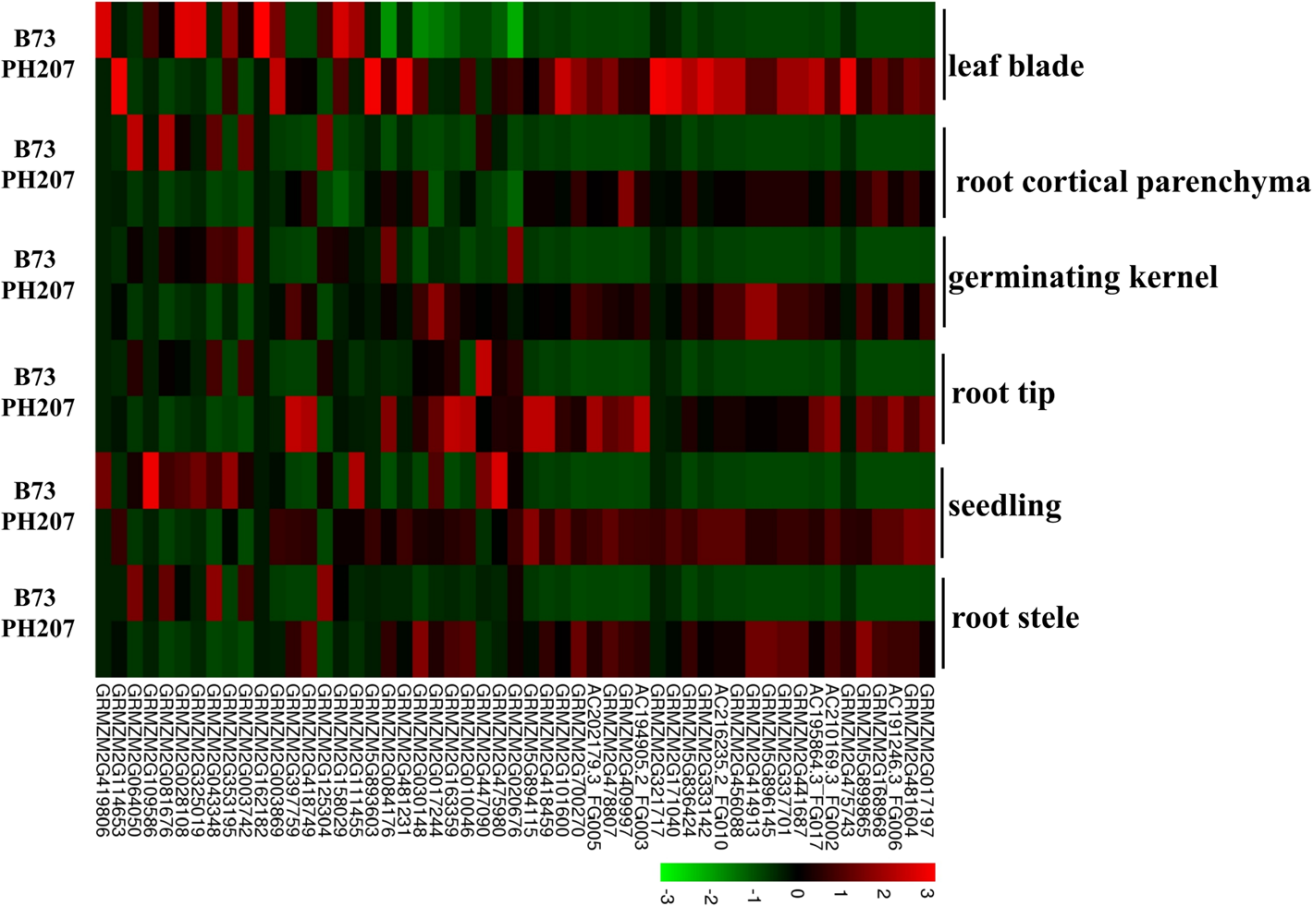
Qualitative expression variation of maize PPR genes in six different tissues

To explore the expression variation of the PPR gene family in B73 and PH207, we collected public expression data for six different tissues (leaf blade, root cortical parenchyma, germinating kernel, root tip, seedling, and root stele) of B73 and PH207. Among these PPR genes, 53 PPR genes exhibited qualitative expression variation in the different genetic backgrounds (Fig. 6). For example, 26 PPR genes not expressed in the six tissues (leaf blade, root cortical parenchyma, germinating kernel, root tip, seedling, and root stele) in the B73 background were expressed in all six tissues in the PH207 genome, and 6 PPR genes that were not expressed in the six tissues in the PH207 background were expressed in all six tissues in the B73 background; the other 20 PPR genes also exhibited distinct expression patterns in the different backgrounds (Additional file 13: Figure S5, Additional file 14: Table S9). We further found that one gene (GRMZM2G162182) was expressed in only the leaf blade in the B73 background (Additional file 14: Table S9). This significant qualitative variation in the expression of PPR gene family members in different genetic backgrounds increases the potential versatility of the biological functions of these genes.

### PPR genes play an important role in maize kernel development

To explore the potential functions of the PPR genes in maize kernel development, expression of the maize PPR genes was analysed in kernels on different days after pollination. Among of the 491 PPR genes in the B73 genome, most of the PPR genes (446) were expressed in kernels (Additional file 15: Figure S6). The statistical results for the FPKM values revealed that these PPR genes exhibited low expression (Additional file 16: Table S10), with only one gene (GRMZM2G110952) exhibiting a high FPKM (fragments per kilobase of exon per million fragments mapped) value (> 100) in the different stages of maize kernel development.

**Fig. 7:**
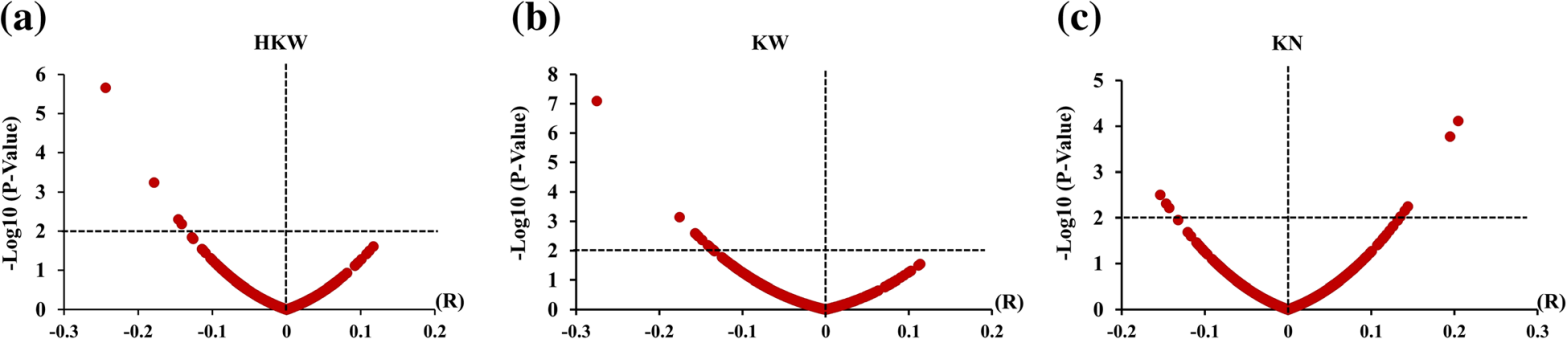
Correlation analysis between expression of the PPR genes in kernels and kernel-related traits. The horizontal coordinate represents the correlation coefficient, and the vertical coordinate represents the -log_10_ value (*P*-value). **a**, **b**, **c** show the correlation with HKW (hundred kernel weight), KW (kernel width) and KN (kernel number), respectively

Furthermore, we collected expression data for PPR genes in kernels from different maize inbred lines (http://www.maizego.org/) to explore correlation between expression of PPR genes in kernels and kernel-related traits, such as hundred kernel weight (HKW), kernel width (KW) and kernel number per row (KN). Four PPR genes, located on chromosomes 1, 2, 7, and 8, were found to be significantly negatively correlated with HKW at the *P* < 0.01 level (Fig. 7a). A total of 12 PPR genes were significantly negatively correlated with KW (Fig. 7b). Additionally, five PPR genes were significantly positively correlated with KN, and three other PPR genes were negatively correlated with KN (Fig. 7c). We also found that some PPR genes are located in the metaQTL region associated with yield and kernel-related traits in maize [27]. For example, GRMZM2G177894, which was significantly correlated with KW, is located in the MQTL-33 region, GRMZM2G021303 is located in the MQTL-46 region, and GRMZM2G123959 is located in the MQTL-27 region (Additional file 17: Table S11). These results suggest that these PPR genes can be regarded as candidate genes that are related to maize kernel development.

**Fig. 8:**
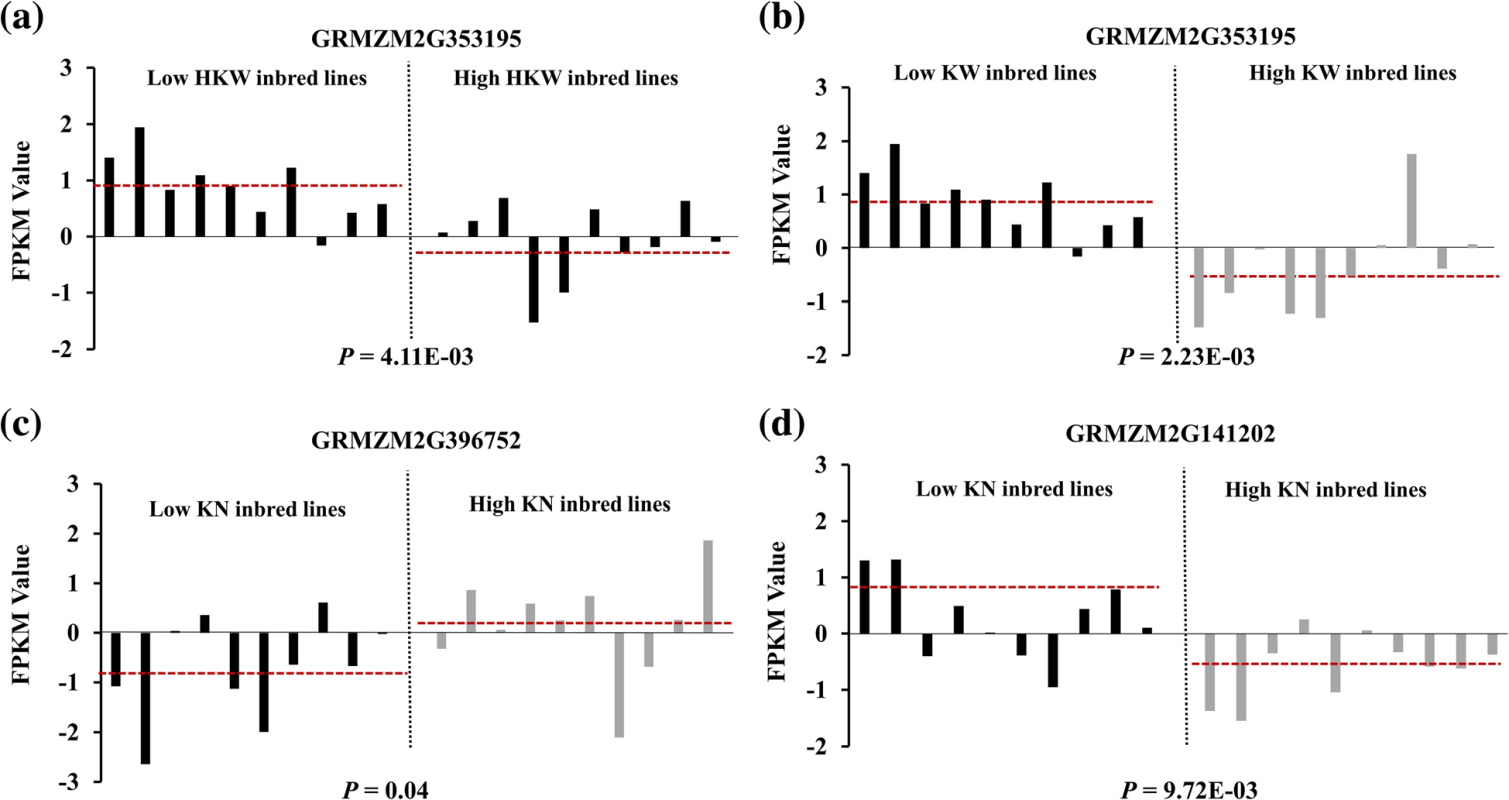
Expression levels of candidate genes in the kernels of small-grain and large-grain inbred lines. **a**, **b** Expression levels of GRMZM2G353195 in high-HKW inbred lines and low-HKW inbred lines and in high-KW inbred lines and low-KW inbred lines, respectively. **c**, **d** Expression levels of GRMZM2G396752 and GRMZM2G141202 in low-KN and high-KN inbred lines, respectively

To further validate the related gene functions, we analysed the expression levels of these candidate genes in kernels from small-grain and large-grain inbred lines and found that expression of GRMZM2G353195 was significantly correlated with HKW and KW, suggesting that GRMZM2G353195 may be pleiotropic (Fig. 8a, b). GRMZM2G396752, which is located in the MQTL-50 region, showed a slight difference in expression between low-KN and high-KN inbred lines (Fig. 8c); GRMZM2G141202, located in the MQTL-43 region, displayed a significant expression difference (Fig. 8d). These results suggest that the GRMZM2G353195 and GRMZM2G141202 PPR protein-encoding genes can be regarded as important candidate genes for maize kernel-related traits.

## Discussion

PPR genes, a plant-specific gene family widespread in higher plants, are reported to be involved in many critical development processes [28]. Systematic and integrative analyses of PPR genes have been performed in *Arabidopsis*, rice and foxtail millet [2–4]. However, little is known about the maize PPR gene family. Hence, in this study, we performed genome-wide analyses of PPR genes in two maize inbred lines with significantly different pedigrees by combining bioinformatic and expression analyses to reveal their important roles during kernel development.

We identified 491 and 456 PPR genes in B73 and PH207, respectively, and these genes were divided into five subgroups. Although the maize genome is larger than that of *Arabidopsis*, rice and foxtail millet, these numbers of PPR genes are very similar to the numbers found in these other species and even less than in some species with small genome sizes [2–4]. This phenomenon can also be observed for other gene families, such as the IQD gene family, which has 26 members in maize [29] and 33 in *Arabidopsis* [30], the ANK gene family in maize (71 members) [31] and *Arabidopsis* (105 members) [32], and the bglu gene family in maize (26 members) [33] and *Arabidopsis* (47 members) [34]. The reason for this may be the fewer gene duplications occurring in the maize genome [35]. The genome of *Arabidopsis* has experienced four extensive duplication events during evolution [36, 37], whereas maize has experienced only two rounds of genome duplication [35]. Interestingly, we found different numbers of PPR genes in the two different maize inbred lines B73 and PH207. Many studies have confirmed the presence of copy number and presence/absence variations between inbred lines [22–24]. For example, a 2.6-Mb region in a chromosome is present in B73 but absent in Mo17 [22]. In an expanded panel of elite maize inbred lines, hundreds of genes exhibit presence/absence variations, showing heterotic group specificity [23, 24]. B73 and PH207 are representative inbred lines from the stiff stalk and the Iodent germplasm groups of maize, respectively [38]. Although numerous structural variants exist between the B73 and PH207 genomes, a few large gaps can identified [26]. Across the whole genomes, 1169 genes are B73 genotype specific, and 1545 genes are PH207 genotype specific [26]. In our study, we found different numbers of PPR genes in the two different maize inbred lines: B73 has 491 PPR genes, and PH207 has 456 PPR genes. Among these genotype-specific genes, there are five PPR genes in the two genomes that might be due to presence/absence variations between the B73 and PH207 genomes.

The absence of introns or the presence of few introns is an important characteristic of the PPR gene family [27, 39]. In *Arabidopsis*, more than 80% of PPR genes contain only a single exon, and only 7% contain more than one intron [2]. A similar pattern is found in rice and foxtail millet [3, 4]. Additionally, in the moss genome, 80% of PPR sequences contain one or more introns [40]. In this study, 78.41 and 62.94% of the PPR genes in the B73 and PH207 genomes, respectively, were predicted to contain only one or no introns. From an evolutionary perspective, previous studies have suggested that intron-rich PPR genes may be ancient genes in the PPR family [2, 4, 28]. These results provide evidence that a majority of intron-poor PPR genes exist in higher plants and originated from intron-rich PPR genes through reverse transcriptional transposition events [40, 41]. In this study, we found one PPR gene (GRMZM2G327263) located on chromosome 3 in the B73 genome that contains 24 introns; in the PH207 genome, Zm00008a017909, located on chromosome 4, contains 17 introns. PPR proteins participate in many biological processes and in plant growth regulation in a range of plant species [42–44].

According to GO analysis, 105 PPR proteins in maize are predicted to be related to metabolic processes, 102 PPR proteins to cellular processes, and 78 PPR proteins to single-organism processes. We found 27 PPR proteins in maize predicted to be responsive to stimuli, suggesting that some PPR genes function in stress tolerance, as shown by previous studies in other species. For example, SOAR1, a PPR protein in *Arabidopsis*, enhances tolerance to abiotic stresses by regulating abscisic acid signalling in seed germination and post-germination growth [45]. SLG1, another PPR protein, can improve drought stress tolerance in *Arabidopsis* [46], and overexpression of the *PPR40* gene improves salt tolerance by reducing oxidative damage in *Arabidopsis* [47]. Microarray data have revealed that 92 PPR proteins of the E subgroup in *Arabidopsis* are differentially expressed under stress treatments [48]. Expression analysis in foxtail millet showed 24 SiPPR genes to be responsive to abiotic stresses [4]. In addition, many PPR proteins can edit the introns of mitochondrial and chloroplast genes to affect plant development in maize and *Arabidopsis* [49–51]. The GO analysis results from this study also predict that many PPR genes have RNA-binding functions, corroborating results in other species.

Analysis of conserved protein motifs in the PPR gene family was also conducted. Although these motifs are not the same as the motifs used to classify PPR proteins into different subgroups, we still found that a majority of the PPR genes from the same subgroup exhibited a similar motif distribution. For example, six motifs were mainly found in the P subgroup; Motif 5 was mainly present in the DYW subgroup, the E subgroup did not exhibit Motif 18, and the PLS subgroup contains all the identified motifs in the B73 genome. Comparison of the results of motif analysis for the PPR proteins in the B73 genome revealed that some are conserved in the two genomes, such as Motif 2 in the B73 genome (Motif 6 in the PH207 genome), Motif 8 in the B73 genome (Motif 9 in the PH207 genome), and Motif 11 in the B73 genome (Motif 2 in the PH207 genome). These conserved motifs may be the essential components that determine the common molecular functions of PPR genes in different subgroups and even in different maize inbred lines.

The embryo, endosperm and surrounding maternal tissues are closely associated with final kernel size or shape [52]. Many genes that are related to kernel size through regulation of embryo and endosperm development have been cloned in maize. Furthermore, PPR genes play an important role in kernel development in maize, including *dek2* [11], *dek10* [53], *dek35* [17], *dek36* [12], *dek37* [54], *dek39* [13], *empty pericarp4* [55], *empty pericarp 10* [10], *empty pericarp11* [18], *PPR8522* [8] and *small kernel 1* [20]. Mutants of these genes often show a delay in embryo and endosperm development and eventually produce a small kernel. Therefore, normal expression of related PPR genes is important to maintain normal kernel development in maize. In this study, we found that a majority of PPR genes are continuously expressed at different stages of kernel development in maize. Through correlation analysis, we successfully identified several PPR genes associated with kernel-related traits. For example, GRMZM2G353195 and GRMZM2G141202 might be regarded as important candidate genes associated with maize kernel-related traits with functions that are worth investigating through a reverse genetics approach. Taken together, our results provide a more comprehensive understanding of PPR genes in different maize inbred lines and provide important candidate genes related to kernel development for subsequent functional validation in maize.

## Conclusion

In this study, 491 and 456 PPR genes were identified in the B73 and PH207 genomes, respectively. Basic bioinformatics analyses, including classification, gene structure, chromosomal location and conserved motif analysis, were conducted. The PPR gene duplication analyses showed that 12 and 15 segmentally duplicated gene pairs exist in the B73 and PH207 genomes, respectively, eight of which are shared. Expression analysis suggested that 53 PPR genes exhibit qualitative variation in these different genetic backgrounds. In addition, analysis of the correlation between PPR gene expression in kernel and kernel-related traits showed that 4 PPR genes are significantly negatively correlated with HKW, 12 PPR genes are significantly negatively correlated with KW, and 8 PPR genes are significantly correlated with KN. Eight of these 24 PPR genes are located in the metaQTL region associated with yield and kernel-related traits in maize. Two important PPR genes (GRMZM2G353195 and GRMZM2G141202) can be regarded as important candidate genes associated with maize kernel-related traits. Our results provide a more comprehensive understanding of PPR genes in different maize inbred lines and provide important candidate genes related to kernel development for subsequent functional validation.

## Methods

### Identification of PPR genes in the maize genome

The PPR gene family motif seed file (PF01535) constructed based on the hidden Markov model (HMM) was downloaded from the Pfam v31.0 database (http://pfam.xfam.org/). The motif file was then used to query the B73 Ensembl-30 (ftp://ftp.ensemblgenomes.org/pub/plants/release-30/fasta/zea_mays/dna) and PH207 genomes (https://phytozome.jgi.doe.gov) [56] with the HMMER 3.0 program (http://www.ebi.ac.uk/Tools/hmmer/) applying an E-value < 10 [57]. The protein domains of the resulting candidate PPR genes in both genomes were analysed by using the SMART program (http://smart.embl-heidelberg.de/). The conserved sequence domains of the PPR gene subgroup used in this study were identified in previous studies based on a range of plant species (*Arabidopsis* and rice). Finally, we used the HMMER matrix defined by the conserved domains of the PPR gene subgroups (P, PLS, E, E+ and DYW) to retrieve, analyse and categorize these protein sequence domains.

### Chromosomal locations, gene structure, genomic distribution and subcellular localization prediction

Detailed information, including chromosomal location, start site information, and lengths, of the PPR protein sequences in the B73 and PH207 genomes can be queried and obtained from the *Zea mays B73 Ensembl-30* and *Zea mays PH207 v1.1* maize databases. We downloaded the genomic sequences and corresponding coding sequences and used Gene Structure Display Server 2.0 software (http://gsds.cbi.pku.edu.cn/) to illustrate the gene structures and statistical intron numbers of these maize PPR genes [58]. According to the physical locations of these PPR genes, we illustrated the distribution of these genes in the B73 and PH207 genomes using Genomepixelizer software [59]. The signal peptide sequence prediction program TargetP (http://www.cbs.dtu.dk/services/TargetP/) [60] was used to predict the N terminal signal peptides for all of the PPR protein sequences in B73 and PH207.

### Gene ontology (GO) analysis and motif identification

We conducted functional annotation analysis of the PPR gene family in B73 and PH207 using the Blast2GO program (http://www.blast2go.com)**.** MEME Suite (http://meme-suite.org/) was employed to identify the motifs of the PPR protein sequences [61]. We used the following parameters to perform the analysis: width of the motif, 8–50; and maximum number of motifs, 19.

### Duplication analysis of PPR genes

Duplication analysis was performed with MCScanX software using the PPR protein sequences and the position data in the genome and was visualized in Circos 0.67. The protein sequences from segmentally duplicated gene pairs were aligned using the software DNAMAN. The PAL2NAL program (http://www.bork.embl.de/pal2nal) was applied to estimate the rates of synonymous (Ks) and nonsynonymous (Ka) substitutions and the ratio of Ka/Ks.

### Expression analysis

Genome-wide gene expression data from the maize inbred lines B73 and PH207 that have been published based on previous studies are useful for illustrating PPR family expression patterns in different developmental tissues and stages of maize [26]. To better analyse PPR gene expression patterns in different genetic backgrounds, we selected transcriptome data that were published from the same study [26] and downloaded such data for six different tissues (leaf blade, root cortical parenchyma, germinating kernel, root tip, seedling, and root stele) of B73 and PH207 from the Dryad repository (10.5061/dryad.8vj84). In this study, we focused on only qualitative variations between PPR genes in the six investigated tissues from B73 and PH207.

At 8, 10, 12, and 14 days post-self-pollination, kernels from the middle of three independent ears were collected from B73 and used to extract total RNA. We presented the RNA sequence data in a previous report [62]. To explore correlation between expression of PPR genes and kernel-related traits (HKW, KW, and KN), we collected data on PPR gene expression profiles in kernels and the kernel phenotype of the association panel, which consists of 368 different maize inbred lines (http://www.maizego.org). The related methods have been described in previous reports [63, 64]. The association panel was planted in Jingzhou in Hubei Province of China in 2010, and immature kernels were collected at 15 DAP (days after pollination) to conduct RNA-sequencing [63]. The kernel-related traits of the association panel were evaluated in five different environments [64]. We further analysed important candidate genes according to correlation analysis, selected 20 maize inbred lines with extreme phenotypes (HKW, KN, and KW), and calculated differences in expression profiles using the t-test procedure in SAS software (Release 9.1.3; SAS Institute, Cary, NC).

## Additional files

Additional file 1: **Table S1.** Detailed information for PPR genes of the B73 genome. (XLS 95 kb)

Additional file 2: **Table S2.** Detailed information for PPR genes of the PH207 genome. (XLSX 90 kb)

Additional file 3: **Figure S1.** Example of the gene loss caused by CNVs in the two genomes. (TIF 369 kb)

Additional file 4: **Table S3.** Shared PPR genes with only one copy in the B73 and PH207 genomes. (XLSX 68 kb)

Additional file 5: **Table S4.** PPR genes from the B73 genome with multiple copies in the PH207 genome. (XLSX 84 kb)

Additional file 6: **Figure S2.** Gene structures of PPR genes in the B73 genome. (PDF 2960 kb)

Additional file 7: **Figure S3.** Gene structures of PPR genes in the PH207 genome. (PDF 2934 kb)

Additional file 8: **Table S5.** Statistical information for GO analysis results for PPR genes in the B73 and PH207 genomes. (XLSX 32 kb)

Additional file 9: **Figure S4.** Detailed GO analysis results for maize PPR proteins in PH207. (TIF 928 kb)

Additional file 10: **Table S6.** Segmental duplications of PPR genes in the two genomes of B73 and PH207. (XLSX 14 kb)

Additional file 11: **Table S7.** Paralogs of PPR genes that are located on the same chromosome. (XLSX 16 kb)

Additional file 12: **Table S8.** Paralogs of PPR genes that are located on different chromosomes. (XLSX 9 kb)

Additional file 13: **Figure S5.** Number of PPR genes that result in qualitative variation based on expression analysis in six different tissues in the B73 and PH207 genetic backgrounds. Numbers in brackets indicate the number of tissues in which PPR genes are expressed. (TIF 453 kb)

Additional file 14: **Table S9.** List of PPR genes associated with qualitative variation in different genetic backgrounds. (XLSX 44 kb)

Additional file 15: **Figure S6.** Expression profiles of PPR genes at different stages of kernel development. (TIF 746 kb)

Additional file 16: **Table S10.** FPKM statistics for PPR genes in developing kernels. (XLSX 8 kb)

Additional file 17: **Table S11.** List of PPR genes associated with kernel-related traits. (XLSX 11 kb)

## Abbreviations

CNVs: Copy number variations
FPKM: Fragments per kilobase of exon per million fragments mapped
GO: Gene ontology
HKW: Hundred kernel weight
Ka: The ratio of the number of nonsynonymous substitutions per non-Synonymous site
KN: Kernel number per row
Ks: The ratio of the number of synonymous substitutions per synonymous site
KW: Kernel width
PAVs: Presence/absence variations
PLS: P-L-S, PPR-like S (for short) and PPR-like L (for long)
PPR: Pentatricopeptide repeat
