# Supplementary figures and images for: Genome-wide analysis of the pentatricopeptide repeat gene family in different maize genomes and its important role in kernel development
Source: BMC Plant Biol. 2018 Dec 19;18:366. doi: 10.1186/s12870-018-1572-2 (PMC6299966; doi:10.1186/s12870-018-1572-2)

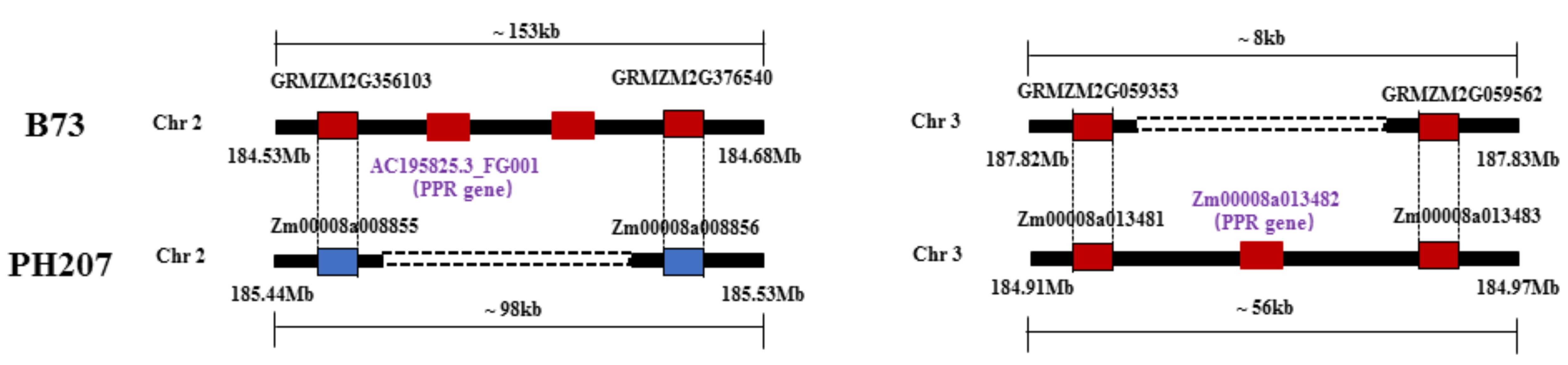

Supplement: Supplementary file 3 — Figure S1. Example of the gene loss caused by CNVs in the two genomes. (TIF 369 kb) [file 12870_2018_1572_MOESM3_ESM.tif]

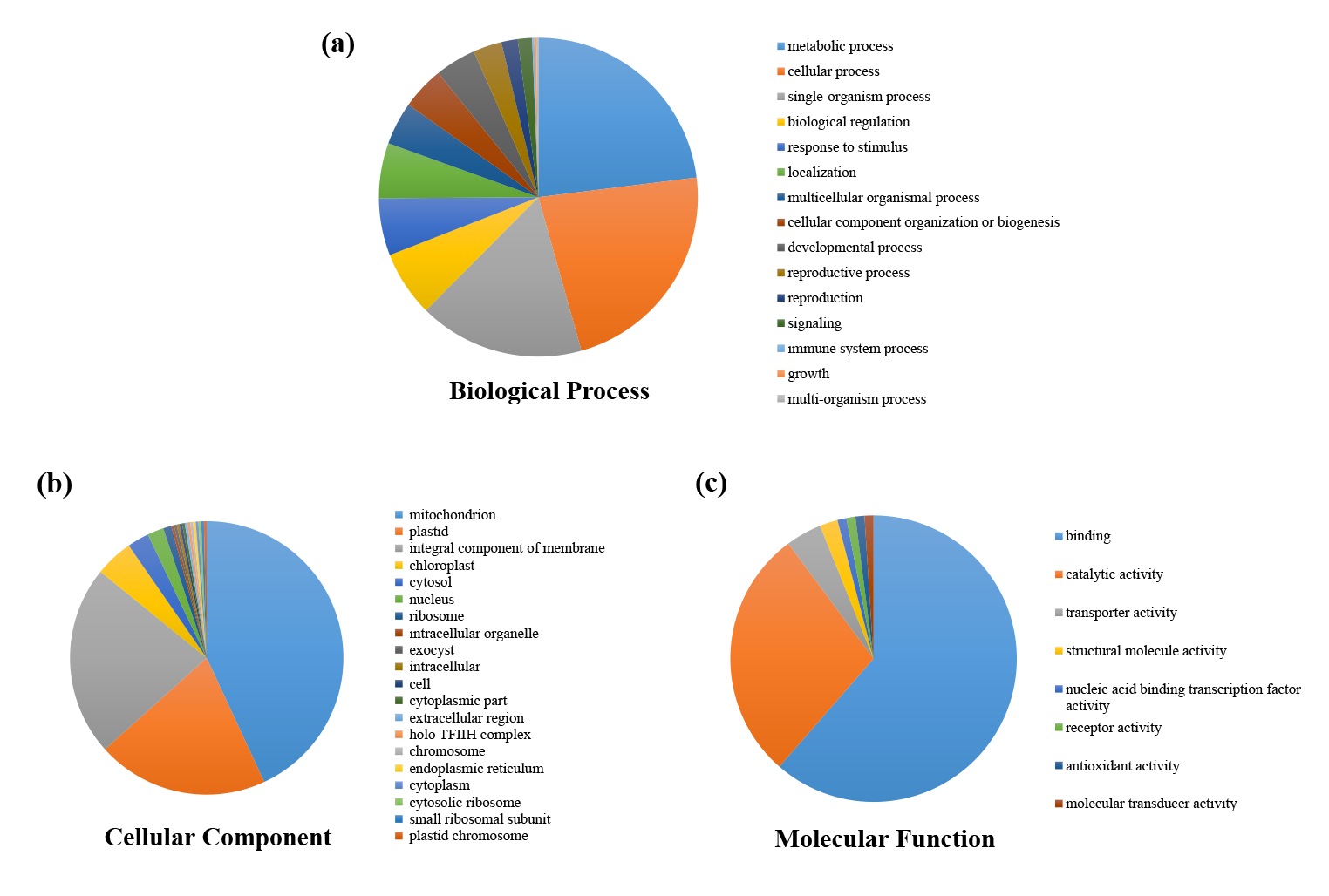

Supplement: Supplementary file 9 — Figure S4. Detailed GO analysis results for maize PPR proteins in PH207. (TIF 928 kb) [file 12870_2018_1572_MOESM9_ESM.tif]

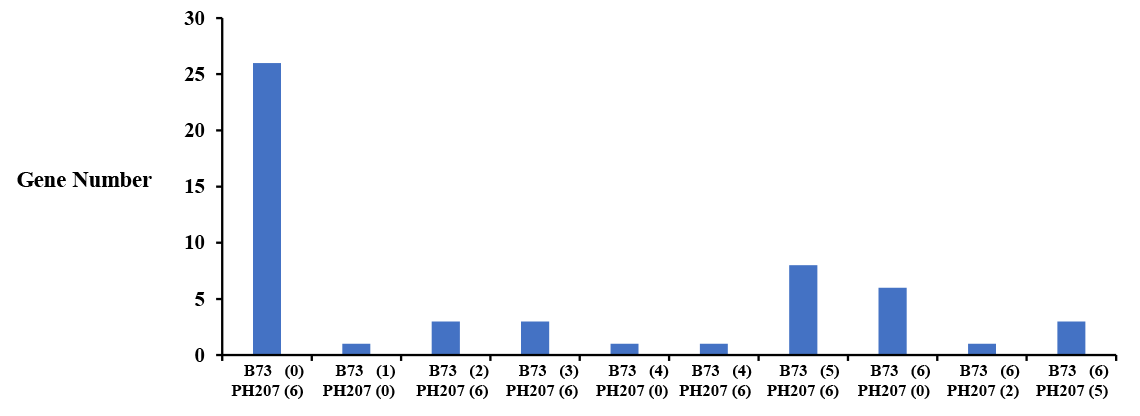

Supplement: Supplementary file 13 — Figure S5. Number of PPR genes that result in qualitative variation based on expression analysis in six different tissues in the B73 and PH207 genetic backgrounds. Numbers in brackets indicate the number of tissues in which PPR genes are expressed. (TIF 453 kb) [file 12870_2018_1572_MOESM13_ESM.tif]

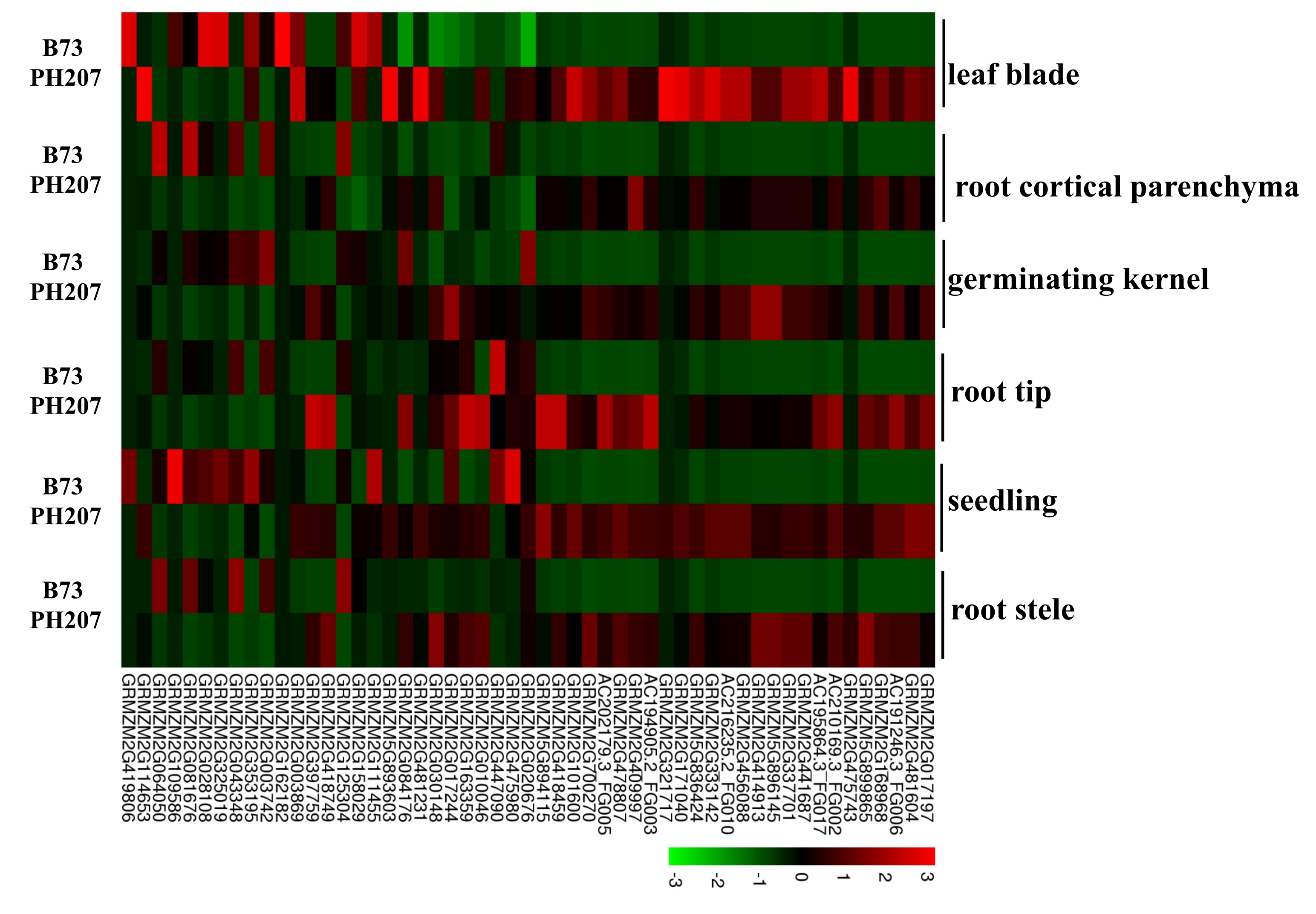

Supplement: Supplementary file 15 — Figure S6. Expression profiles of PPR genes at different stages of kernel development. (TIF 746 kb) [file 12870_2018_1572_MOESM15_ESM.tif]
