# Supplementary material for: Genome-wide analysis of the pentatricopeptide repeat gene family in different maize genomes and its important role in kernel development
Source: BMC Plant Biol. 2018 Dec 19;18:366. doi: 10.1186/s12870-018-1572-2 (PMC6299966; doi:10.1186/s12870-018-1572-2)
Supplement: Supplementary file 6 — Figure S2. Gene structures of PPR genes in the B73 genome. (PDF 2960 kb) [file 12870_2018_1572_MOESM6_ESM.pdf]

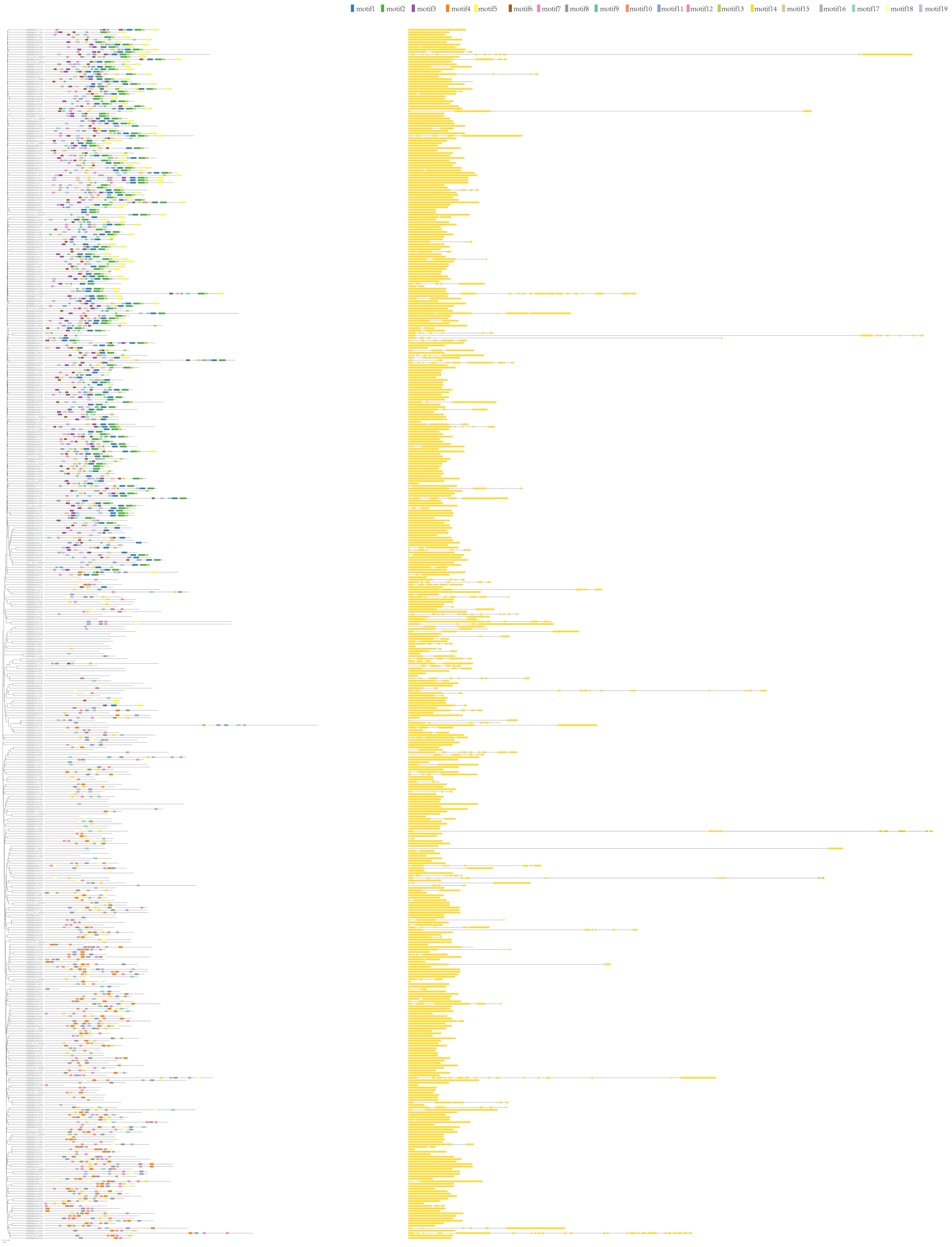
motif1
motif2
motif3
motif4
motif5
motif6
motif7
motif8
motif9
motif10
motif11
motif12
motif13
motif14
motif15
motif16
motif17
motif18
motif19
